# From Tones in Tinnitus to Sensed Social Interaction in Schizophrenia: How Understanding Cortical Organization Can Inform the Study of Hallucinations and Psychosis

**DOI:** 10.1093/schbul/sbu041

**Published:** 2014-06-13

**Authors:** Dominic H. ffytche, Cynthia G. Wible

**Affiliations:** ^1^Department of Old Age Psychiatry, Institute of Psychiatry, King’s College London, De Crespigny Park, London, UK;; ^2^Harvard Medical School, VA Boston Healthcare System, Brockton, MA

**Keywords:** temporal parietal, junction, heteromodal, cortex, unimodal, cortex, multimodality, hallucination, network

## Abstract

The content, modality, and perceptual attributes of hallucinations and other psychotic symptoms may be related to neural representation at a single cell and population level in the cerebral cortex. A brief survey of some principles and examples of cortical representation and organization will be presented together with evidence for a correspondence between the neurobiology of brain areas activated at the time of a hallucination and the content of the corresponding hallucinatory and psychotic experiences. Contrasting the hallucinations of schizophrenia with other conditions, we highlight phenomenological aspects of hallucinations that are ignored in clinical practice but carry potentially important information about the brain regions and dysfunctions underlying them. Knowledge of cortical representation and organization are being used to develop animal models of hallucination and to test treatments that are now beginning to translate to the clinical domain.

## Introduction

Brain imaging has made substantial contributions to our understanding of hallucinations. One advance has been in the characterization of brain changes that predispose an individual to having hallucinations (hallucination trait); these have helped delineate structural and functional changes in brain networks of those at risk of hallucinations in schizophrenia, Parkinson’s disease, and the dementias (see Allen et al^1^ and ffytche^2^ for reviews). Our focus here is on a different aspect of hallucination neurobiology, the activity captured at the time of a hallucination (hallucinating state) and its relationship to hallucination content, a domain of psychopathology that has received little research attention in the modern era. The identification of cortical regions activated during a hallucination helps categorize the experience in terms of its relationship to memory, perception, imagery, emotional circuitry, and better understand the consequences of trait changes in brain networks. However, our ambition goes beyond this in an attempt to fully explain the content of hallucinated experience in terms of the underlying cortical regions. By matching the location of activation foci during hallucinations to regions with putative homology to those identified in nonhuman primate studies of neurophysiological properties at the single cell or neuronal population level, it is apparent that hallucination content and cortical specialization are directly related. For simple visual and auditory experiences at least, evidence is emerging that the content of a hallucination is defined by the single cell and population properties of cells activated at the time of the hallucination. Put another way, the phenomenological content (or qualia) of hallucinated perceptual experience is linked to the neurobiology of cortex activated at the time of the hallucination.

Here, we review the evidence linking cell properties and perceptual content as a starting point for a theory of how cortical specialization may help understand hallucination content in schizophrenia and other experiential aspects of the disease.^3^ Neuroanatomical and electrophysiological evidence has led to a greater understanding of basic properties of perception (eg, the computation of size, shape, and color constancy) and, more recently, of conceptual psychological functions such as agency, intention, insight, social prediction, and self-representation. We will describe the relationship between lower level perceptual content and these higher level conceptual functions.^4–8^ Our hope is that the same type of evidence can be used to develop our understanding of psychopathology and that, in turn, the psychopathology can help our understanding of the underlying neurobiological mechanisms. Just as perceptual content is used to identify disease processes in neurological disorders such as epilepsy—where the semiology of a seizure points to its cortical location—our aspiration is that perceptual content of hallucinations will inform neurobiological accounts of psychiatric disease. The work is based on a presentation and workgroup discussion at the 2nd International Consortium on Hallucination Research meeting, Durham, 2013.^3^

## A General Overview of Sensory Representation

Representation refers to the response properties of a neuron. The cortex contains, in many areas, small functional maps containing neurons with similar response properties. Sensory cortex is generally organized in a hierarchical manner. Primary regions have a fixed structure and represent simple features. Higher order regions lack a rigid structure and respond to complex combinations of features and real-world stimuli (especially for hearing, touch, and vision). Although cortical processing is hierarchical, it is likely to be recursive and proceed in a parallel fashion. Object perception may be supported by activity in small functional regions or by distributed activity over a number of related functional regions.^9^

**Fig. 1.**
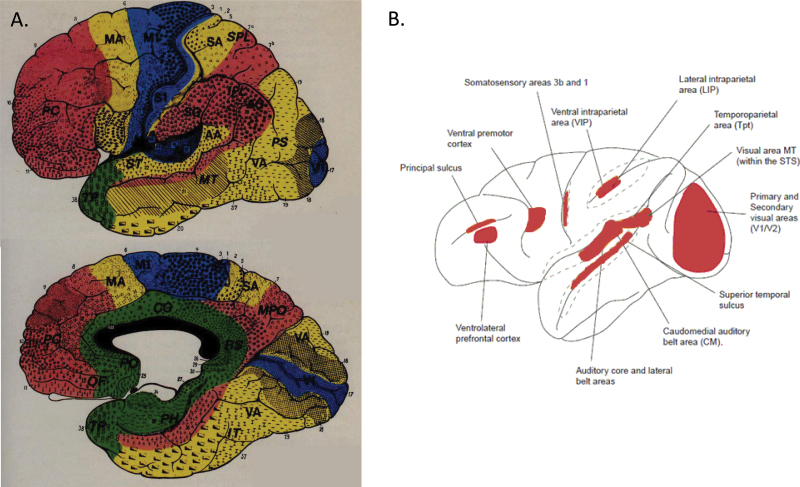
Hierarchical cortical organization. (A) Figure adapted from Mesulam.^13^ A schematic of cortical organization showing primary sensory/motor cortex (blue), unimodal cortex (yellow), and heteromodal (multimodal—red) superimposed on a map of Brodmann areas. (B) Figure adapted from Ghazanfar.^15^ Areas exhibiting cross-sensory interactions are shown in red, superimposed on a schematic of the nonhuman primate brain. The map is similar to that shown in (A) with the addition of primary sensory areas.

The hierarchical representations of visual, auditory, and somatosensory systems remain separate in unimodal association cortex, first converging in heteromodal association cortex where neighboring neurons may respond to different senses or individual neurons respond to more than one sense.^10–12^ The same hierarchical scheme applies to the motor system with low-level motor actions represented in primary motor cortex, the representation of more complex movements in higher tier areas, and planning and sequencing and heteromodal representation in prefrontal cortex. Figure 1A ^13^ illustrates the approximate location of primary cortex and higher order cortex (unimodal and heteromodal cortex) based on cytoarchitectonic, neurophysiological, and clinic-pathological correlation evidence. Primary cortex in each sense (blue) is surrounded by unimodal cortex (yellow) with heteromodal cortex (red) at the junction of different unimodal systems.^13^ Functional imaging evidence suggests heteromodal cortex may be larger than noted on figure 1A,^13^ especially in lateral temporal regions.^11^ Recent evidence has highlighted cross-sensory interactions at the earliest levels of sensory representation (eg, sounds affecting visual processing in primary visual cortex or touch affecting auditory processing in primary auditory cortex), extending the regions in which senses are combined beyond heteromodal cortex (figure 1B).^14,15^ However, this is not to say that neurons in auditory cortex respond to a visual stimulus presented in isolation. The evidence demonstrates that the response to an auditory stimulus in auditory cortex may be modulated by a simultaneously presented visual stimulus. This distinction is of particular importance to our understanding of the neurobiology of hallucination content. In unimodal cortex, although it may be modulated by other senses, the representation is of a single sense modality.

**Fig. 2.**
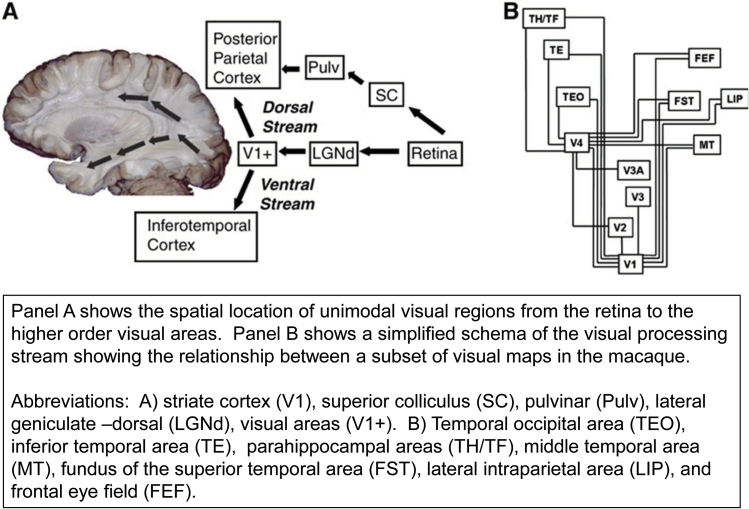
Visual areas from Wandell.^17^

Details of the hierarchical scheme presented above are best understood for the visual system (see Zeki^16^and Wandell et al^17^ and figure 2), but auditory and tactile systems are organized along similar lines. Visual information is first processed in V1, or striate cortex, although direct inputs to motion specialized regions on the lateral occipital surface by-pass V1.^18–21^ V1 neurons have a retinotopic organization and small receptive fields (neighboring regions of the image are represented by neighboring neurons with the right hemifield represented in the left hemisphere and vice versa), and they respond to simple visual features such as orientation, spatial frequency, size, color, and simple texture.^17,22^ Visual processing proceeds through several stages that converge in the temporal cortex and parietal lobe. Inferotemporal neurons have large bilateral receptive fields that include the fovea (center of attention) and respond to complex stimuli such as objects or a monkey hand.^23,24^ The neuronal firing also shows constancy with respect to size (distance), color or illumination, partial occlusion, and object articulation.^25–27^ Hence, in higher order unimodal visual cortex, the convergence of information from the visual processing stream creates representations that convey the information necessary for real-world perception.^28^

**Fig. 3.**
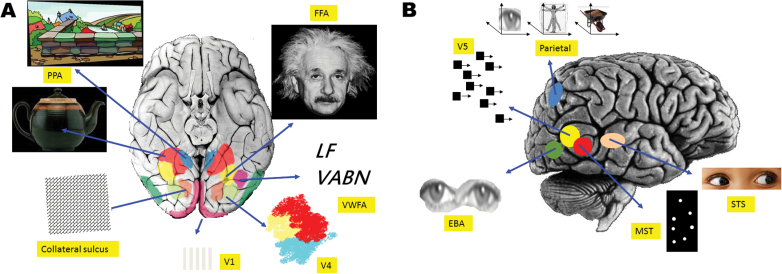
Visual cortical specializations. (A) Ventral view of the brain. Selected areas of relative specialization for (from bottom clockwise) contrast, orientation, and luminance (V1); visual texture patterns; objects; landscapes (PPA, parahippocampal place area); familiar faces (FFA, fusiform face area); text and letter strings (VWFA, visual word form area); and colors V4. (B) Lateral view of the brain. Specializations (from left bottom clockwise) for body parts (EBA, extrastriate body part area), motion (V5), parietal eye, body and object reference frames, gaze and social eye signals (STS, superior temporal sulcus), and biological motion (MST).

The inferior and ventral temporal cortex of humans, most notably the fusiform gyrus, also contains regions that preferentially respond to complex visual features such as text, faces, bodies, and places, see figure 3A.^29^ The visual parietal cortex has representations for space in different coordinate systems (eg, eye-centered, body-centered and object-centered)^30,31^ which, in some areas, remap in preparation for eye movements (figure 3B). The region is thought to have a role in the perception of space, reaching, attention, and salience.

Although details of the auditory system and its homology with the human brain is less understood, particularly in relation to language that is not amenable to study in nonhuman primates, a similar hierarchical scheme exists with representations of increasing complexity in higher level auditory areas. As with vision, auditory processing proceeds through cortical regions that represent increasingly complex auditory features. The primary auditory cortex has a tonotopic organization where neurons that are next to each other respond to similar frequencies.^32^ The representation becomes increasingly sophisticated in higher order auditory regions. For speech, phonological word forms may be represented in the middle portion of the superior temporal sulcus (STS)^33^ with, in man, different aspects of language represented in a posterior temporal region (Wernicke’s territory) and an inferior parietal (IP) region (Geschwind’s territory).^34^ The somatosensory system has representations of touch at different body locations in the primary sensory cortex (postcentral gyrus) and representations of more complex sensations (eg, shapes) in unimodal association cortex in the parietal lobe.

The best studied of heteromodal regions in nonhuman primates is the temporal parietal junction (TPJ), here loosely defined as a region encompassing posterior superior temporal sulcus (PSTS), inferior parietal lobule, and lateral temporal/occipital regions. In the TPJ, higher order visual representations make contact with dynamic auditory and somatosensory representations of voice and gestures. Activity in the (right) IP is highly correlated with that of the anterior insula,^35^ which, in conjunction with the IP, is activated during vocal and subvocal speech production and during verbal working memory.^36,37^

## Hallucinations and the Sensory Hierarchy

How does activity captured during hallucinations relate to the hierarchical network described above? If spontaneous activation of a given cortical representation gives rise to a hallucination of that representation,^2,38–40^ one might make a number of predictions. The first is that simple hallucinations in a given modality involve cortical regions at a lower level of the sensory hierarchy than complex hallucinations, and, conversely, complex hallucinations involve cortical regions at a higher level of the hierarchy. Second, hallucinations involving a single modality (referred to clinically as isolated hallucinations) should relate to unimodal cortex, while hallucinations combining modalities (referred to clinically as multimodal hallucinations) should relate to heteromodal cortex or combinations of heteromodal and unimodal cortical activity. Third, even if arising de novo in a region, activity underlying a hallucination takes place within the context of a network and thus may propagate to other regions. Finally, in addition to sensory experience, conceptual psychological aspects of function implicit in heteromodal representation (eg, social interaction) might be incorporated into the overall phenomenology of the experience. Below, we examine the evidence supporting these predictions and their wider implications for hallucinations in schizophrenia.

## Early Processing and Isolated Simple Hallucinations

### Auditory System

Tinnitus, hearing a tone, or buzzing sound is most often the result of peripheral hearing loss. The experience of hearing a “phantom” tone is directly related to the representation found in primary auditory cortex. Two neurophysiological phenomena have been found to be related to tinnitus.^41^ First, hearing loss results in reduced inhibition in neurons that are tuned to the “lost” frequency. This causes an increased propensity for neurons representing the deaf frequency to fire.^41^ The finding that the pitch of tinnitus is in the hearing loss frequency range in humans and animal models suggests that this increased excitability is the source of tinnitus.^41^ Second, the tonotopic map in primary auditory cortex undergoes reorganization with a larger territory and increased responsiveness for the neurons that are devoted to unaffected or hearing frequencies.^42,43^ Whether or not this cortical reorganization can alleviate or cause tinnitus is a matter of debate. However, tinnitus treatments have been developed that are based on these findings. Researchers have used stimulation of the auditory cortex in human subjects to treat tinnitus.^44^ A recent study used stimulation to hasten cortical map reorganization in human subjects.^43^

### Visual System

Stimulation of primary visual cortex during neurosurgery causes simple visual sensations such as seeing a light (phosphene).^45^ Patients with abnormal activity due to occipital seizures reported hallucinations of simple circular spots or balls during the seizure.^46^ In migraine aura (visual symptoms preceding headache), the experience of scintillating white noise followed by a moving patch of blindness (scotoma) has been linked to activity initially in extrastriate cortex (V3A) but spreading slowly over primary visual cortex.^47^

### Somatosensory Systems

Tactile hallucinations range from itches, scratches, and electrical shocks to sensations of infestation and sexual interference^48^ although it is unclear whether this is can be considered variation in tactile complexity. Hallucinations of touch to different regions of the body have been associated with activation of both lower (thalamus and primary somatosensory cortex) and higher somatosensory areas (IP).^49^

## Higher Level Processing and Isolated Complex Hallucinations

### Visual System

Complex visual hallucinations may follow loss of visual input to V1 through eye disease or lesions of afferent visual pathways (Charles Bonnet Syndrome [CBS]).^50,51^ These hallucinations range from simple colors, blobs, and lines through geometrical patterns to complex objects, figures, disembodied faces, text, and letter strings.^52^ A subset of these categories have been captured in imaging studies of patients,^38^ and imaging studies of induced hallucinations in normal-sighted subjects.^2^ Such studies have found a correspondence between the content of a hallucination and the visual specialization of the area activated. For example, hallucinations of color blobs or unfamiliar faces are associated with increased activity in color and face specialized cortex, while hallucinations of patterns are associated with increased activity in ventral occipitotemporal cortex specialized for geometrical forms.^2^ There is also evidence from lesion studies that hallucinations of text with semantic meaning involve anterior areas of the temporal lobe, contrasting with text-like letter strings (orthographic hallucinations) involving the visual word form area in posterior occipitotemporal cortex.^53^ Hallucinations of lights, multicolored patterns and torsos in a patient with bilateral occipital strokes responded to occipital lobe repetitive transcranial magnetic stimulation (rTMS).^54^

A disadvantage of studying visual hallucinations in visual impairment is that the subjects cannot be stimulated visually to confirm the location of cortex specialized for different visual attributes. A case study of activity during visual hallucinations in schizophrenia found a network of areas activated during hallucinations of family members sitting at a table in a familiar room with objects in their hands. Regions within this network were confirmed in separate experiments to be specialized for places and body parts.^39^ The network also included the hippocampus, which may play an important role in the underlying mechanism (see below).

### Auditory System

Musical hallucinations occur in the context of hearing loss, with content ranging from “angelic choirs” to repeating “old time tunes” (the auditory Charles Bonnet Syndrome) and are associated with activation of higher order auditory cortex in the posterior temporal lobes, basal ganglia, cerebellum, and inferior frontal cortex.^55^ A recent meta-analysis of activity at the time of auditory verbal hallucinations (hearing voices) identified a network consisting of Broca’s (and right hemisphere equivalent), Wernicke’s, and Geschwind’s territories;^34^ the left hippocampus/parahippocampal gyrus; bilateral anterior insula; and right basal ganglia.^56^

## Heteromodal Cortex and Multimodal Hallucinations

The evidence presented above relates to activity occurring during hallucinations of a single modality. In schizophrenia, Parkinson’s disease, and the dementias, several types of hallucination can occur in the same individual.^57^ These may be experienced at different times (eg, an auditory hallucination one day and a visual hallucination the next) when they might be considered sequential instances of unimodal hallucinations. However, in some patients, different modalities occur at the same time. These are referred to as multimodal hallucinations.^58^ Such simultaneous sensory experiences may be perceptually unrelated (eg, “seeing the devil” while “hearing the voice of a relative” from a different location) or perceptually combined (eg, the voice comes from the hallucinated figure),^58^ the combined variant thought to be rare in psychosis.^59^ The prevalence of occasional multimodal hallucination in schizophrenia has been estimated as 50%.^60^ An imaging study of multimodality hallucinations in a patient with schizophrenia (moving colored scenes with rolling disembodied heads simultaneously speaking and instructing) found activity in an extended network that included the TPJ, precuneus, posterior cingulate, and parahippocampal gyrus.^61^ Another study described a patient with schizophrenia and hallucinations of disfigured, living dead bodies that spoke to him about mutilating himself. An analysis of the imaging data using hallucination timings based on button press reports identified activity within the superior temporal gyrus, insula, and regions linked to the button press. Independent component analysis of the data also identified activity in the PSTS, fusiform gyrus, and anterior cingulate.^62^ A more recent study of isolated auditory, isolated visual, and multimodal hallucinations in 20 adolescents with psychosis^63^ found visual hallucinations were associated with activation of visual cortices, auditory hallucinations with a network corresponding to Broca’s, Wernicke’s, Geschwind’s territories and multimodality hallucinations with TPJ activation (PSTS, IP, occipitotemporal junction) and anterior insula.

## Insula, Limbic Cortex and Hallucinations

Epileptic foci or stimulation of the human insula produce a number of visceral sensory and motor responses;^64^ consistent with its proposed role in interoception.^36,37,65,66^ Stimulation of the hippocampus and amygdala are associated with complex multimodality dream-like experiences, isolated somatosensory, auditory, visual, visceral hallucinations, déjà vu experiences, emotions, and alterations in consciousness.^67^

## Beyond Localized Activity—Hallucinations Within Networks

Although the evidence above is presented in terms of localized activity, the cortical regions underling hallucinations are situated within cortical networks with the potential for propagation from one region to another. Such propagation is illustrated by the evolving phenomenology of epileptic seizures where a hallucination may progress from tinnitus to the perception of noise and then finally to noises that sound like incomprehensible voices.^68^ This phenomenological progression matches the shift in representation from tones (primary cortex) to the higher order cortical auditory representation of verbal sounds and voices.

**Fig. 4.**
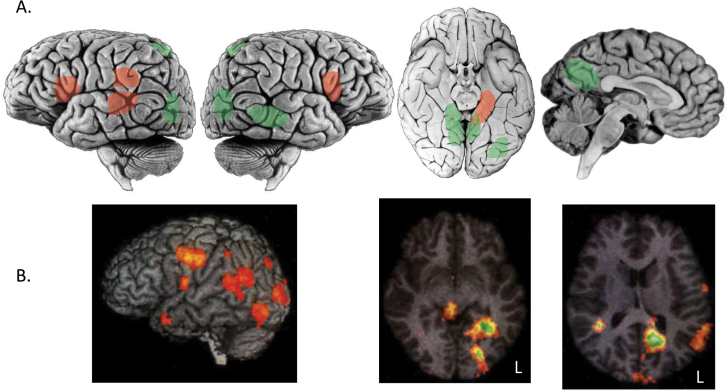
Hallucination network in schizophrenia. (A) Summary of activation during auditory verbal hallucinations (red) or visual hallucinations (green) reported in previous studies^39,56^ from lateral, ventral and medial views of the brain (insula and basal ganglia not shown). (B) Activation during multimodality hallucinations (visual and voice content) adapted from.^61^ Activity is shown in lateral view (left panel) and axial slices at Talairch *z* = −7 (middle panel) and *z* = +13 (right panel).

The importance of networks in the hallucinations of schizophrenia is highlighted in figure 4A,^56,69^ which summarizes the findings of brain imaging studies capturing activity during auditory (red) or visual (green) hallucinations. Both modalities are associated with activation in the hippocampus and TPJ, the difference between the 2 networks being the activation of frontal cortex, insula, and basal ganglia in auditory hallucinations and cuneus/posterior cingulate and ventral occipitotemporal cortex in visual hallucinations. Brain activity during multimodality hallucinations in schizophrenia are an approximate composite of the auditory and visual hallucination networks (figure 4B).^70^ These networks imply connectivity between their constituent regions. This might reflect connectivity at rest in those susceptible to hallucinations; however, evidence of changes in connectivity during visual hallucinations,^2^ suggests the connectivity network may only be apparent at the time of a hallucination.

Whether the networks illustrated reflect activity propagated from aberrant activity in one of the nodes to other parts of the network is unclear. Speech is processed in an obligatory manner such that both the perception of speech and the subvocal reproduction of speech activate a network of sensory and motor regions,^33^ and there is no reason to suspect this might not also be case when activity arises spontaneously in part of the network. Activity in the parahippocampal gyrus decreases immediately prior to auditory hallucinations, suggesting there may be a temporal sequence to the activity.^71^ One possibility is that the primary abnormality in the network illustrated is spontaneous activation of the TPJ, perhaps caused by altered interactions with the hippocampus.^72,73^ This activity is propagated across the network, with the phenomenology of the hallucinated experience depending on the distribution of propagated activity: voices if to frontal cortex and insula, visual if to occipital and ventral temporal cortex, and multimodality if to both. The contribution of the posterior cingulate and medial parietal regions to visual phenomenology is unclear but may relate to their role in hippocampal connectivity or default mode mentation.^74^ This type of account, based on the spontaneous activation of sensory cortex, has been contrasted in a recent review^75^ with traditional accounts linking auditory verbal hallucinations to deficits in self- or source-monitoring, corollary signals,^76,77^ or expectation bias and prediction error.^78^

While the neurophysiological mechanisms underlying the activated network in schizophrenia remain unclear, the network appears to differ from that associated with hallucinations in other conditions. In CBS, activity appears more restricted without the involvement of the hippocampus or frontal regions. Simple hallucinations predominate over complex ones, as the likely result of the greater impact of visual deafferentation on lower levels of the hierarchy than higher level ones.^2^ Although one might expect neurophysiological changes at lower levels to be propagated to the TPJ in CBS resulting in multimodality hallucinations with implied social interaction (see below), for reasons that are unclear, this does not seem to occur.^57^

## Hallucinations and Psychosis

Hallucinations are not synonymous with psychosis. In CBS, hallucinations arise in the absence of other psychopathology and can be examined and appraised as any normal perceptual experience. The same is not true of hallucinations in schizophrenia, which have prominent emotional impact, are incorporated into a delusional system, and patients lack insight into their nature. How does the evidence outlined above account for this difference and the wider psychopathological setting of hallucinations in schizophrenia? As described in the following sections, one possibility is that phenomenological features of hallucinations in psychosis are implicit in aberrant activation of the TPJ.

### Representation of Social Interaction and Its Psychopathology

TPJ neurons or neuronal populations represent voices and faces, facial and body emotional expressions, prosody, and eye gaze (attention) that form the basis of social interaction.^5,8,72,79–83^ The TPJ is the neural substrate for theory of mind.^82,84–86^ Correspondingly, the human TPJ has been shown to be a core region for the perception of live social interaction.^80,87^ The basic or concrete function of the region is the perception of dynamic gestures^88^ in the auditory,^89^ visual, and somatosensory domains; it may constitute a supramodal area that is involved in human motion recognition, irrespective of the sensory modality input.^90^ In primates, auditory-visual representation is the dominant type of representation in the STS.^91^ When audiovisual STS neurons are activated, they in turn produce feedback to excite lower level perceptual regions (especially auditory cortex), resulting in increased activity and gamma band synchronization between the 2 regions.^12^ This feedback is preattentive and occurs in the auditory, visual, and somatosensory domains; it has been found in humans.^92–95^

Along with the concrete sensory representation of dynamic gestures in TPJ, there is an inherent computation of the intention of others as inferred from their actions. Single neuron recordings show that activity in the STS is automatically tuned to predict actions.^6–8,96^ Neuronal recordings in the STS and functional MRI imaging of the TPJ report that this region is poised to quickly and preferentially respond to social threat and fear.^8,97,98^

What type of hallucinated experience might be predicted from aberrant activation of this type of representation? One prediction would be predominance of voices over other types of hallucinatory content, associated with dynamic visual human action^99^ and somatosensory hallucinations. This description closely resembles the known phenomenology of hallucinations in schizophrenia. Auditory verbal hallucinations of voices predominate in schizophrenia over other modalities, but, as described above, voices may be combined with visual or somatosensory hallucinations, either simultaneously or sequentially. Hallucinations in schizophrenia are perceived as arising from others with a false sense of social interaction and of others’ intent that can plausibly be linked to aberrant activation of the TPJ. In support of this view, stimulation of the TPJ causes the feeling of a presence or of being followed by a shadowy person.^100,101^ In one report, the presence was perceived to be trying to interfere with the language testing (“He wants to take the card”).^100^ Paranoid delusions prompted by a hallucination are plausibly linked to implied actions of others in the hallucinated experience, and abnormal activity in the TPJ has been linked to persecutory delusions.^102^ Delusions of control in schizophrenia are related to abnormal activation in the right IP and insula^103–108^

### Representation of the Self, Insight, and Its Psychopathology

**Fig. 5.**
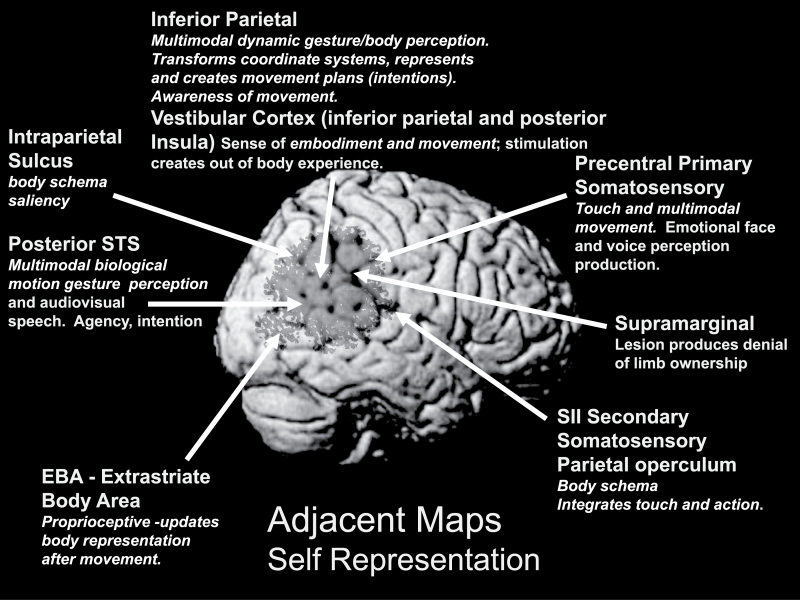
Self-representation in the TPJ, based on Blanke and Arzy.^109^

The perception of self-made gestures from our own visuospatial perspective and the corresponding auditory, visual, tactile, proprioceptive, and vestibular information resulting from actions may form the basis for the feeling of “self” and of being an agent.^109^ These elements are represented within the TPJ (figure 5).^65,79,109–111^ The stages of self-representation and the separate inputs are experienced as a gestalt (or not consciously experienced as separate).^110^ The simple manipulation of these simultaneous convergent inputs (eg, visuospatial perspective and touch) can cause a disturbance in the self-representation and the feeling of embodiment in otherwise psychiatrically healthy individuals.^112–114^ The IP region contains neurons that represent our own intentions to act (premotor movement plans); IP activity also conveys the feeling of executing and being an agent of the movement.^115,116^ The sense of agency is thought to be a result of a combination of internal motoric signals and external sensory evidence about the source of actions. Disruptions in the timing or perceptual strength of these signals can disrupt agency judgments in healthy controls.^117^ Insight is a complex construct with several subcomponents.^118^ One aspect, the sense of ownership of body parts or symptoms, is linked to the IP and insular regions with damage to IP cortex associated with anosognosia (the denial of deficits such as blindness or paralysis). In somatopharaphrenia or verbal asomatognosia, patients (usually with hemiparesis) believe that their limbs are absent or deny ownership of their limbs.^119,120^ Anosognosia for hemiplegia is thought to relate to a dominance of IP motor intention activity prior to action over sensory information about the actual effects of movement.^121^

Disorders of self-perception, as reflected in insight and self-agency, are defining characteristics of schizophrenia and are linked to a range of symptoms including hallucinations, passivity phenomena (abnormal perception of the control and ownership of movements), and thought insertion (eg, thoughts come not from self but from outside the head or television). Lack of agency in schizophrenia could stem from a number of sources,^122^ including the feeling of an external presence overriding the representation of self, a failure to activate or abnormal activation in IP regions^108^ that confer action awareness or abnormal self-representation.^117^ Specific examples of abnormal self-representation in schizophrenia have been described in the literature. For example, Pontius^123^ reported a patient who experienced no body schema or self, only empty space, and Cutting^124^ described individuals who felt a body consisting only of an outside frame, duplicated bodies, and alterations in the size and position of the body parts. Such disorders of the self are consistent with aberrant activity in the TPJ. In CBS, there are transient increases in activity accompanied by an elevated baseline in those regions affected.^125,126^ It is possible that an elevated baseline in the TPJ (and hippocampus) could be present in schizophrenia.^127,128^ Hence, both transient and sustained increases in activity contribute to problems with self and other perception through changes in timing or in the perceptual balance of inputs.^122^ Disorders of self in schizophrenia might also be related to a persistent deficit of TPJ function, analogous to a lesion causing anosognosia.

## Clinical Implications and Future Directions

While a compelling case can be made for linking single cell and population properties of cortical regions with the contents of hallucinations, the evidence presented above leaves open the question of what triggers a given cortical area to activate. Hallucinations in schizophrenia clearly relate to activation of a network with the functions of the TPJ and functional links to the hippocampus,^74,129,130^ indicating that these structures play a key role, irrespective of modality. Networks appear to be less important in CBS so do not account for hallucinations in all disorders. Hallucinations may thus be conceived as a family of symptoms, each with different underlying mechanisms^57^ but sharing a final outcome of activation in sensory cortex.

What are the implications for clinical practice? First, the perspective of cell properties helps divide pathological experience into neurobiologically meaningful categories. The distinction of isolated vs multimodality hallucinations is largely ignored in clinical practice; indeed, the distinction is not captured in many hallucination assessment interviews (eg, Scale for the Assessment of Positive Symptom, Psychotic Symptom Rating Scales, and Positive and Negative Syndrome Scale). However, in terms of brain systems and regions implicated, isolated and multimodal hallucinations are entirely distinct categories. Similarly, with the exception of auditory hallucinations, the relevant content of hallucinations is often not noted (eg, presence or absence of implied social interaction). It has long been recognized that elementary sounds or colors, flashes, and lines have different clinical implications to voices or formed objects; however, little attention has been given to the localizing information implicit in the dimensional axis of simple to complex or socially irrelevant to relevant content. Knowledge of the likely cortical location of a given set of hallucination contents may help decide on whether focal treatments using rTMS^131^ will be helpful or point to specific pharmacological strategies based on the neurotransmitter profile of regions implicated. The recognition of content as a neurobiological signature is an important step toward understanding the mechanisms of hallucinations in different clinical conditions and the development of novel treatment strategies.

## Funding

The Second Meeting of the International Consortium on Hallucination Research^3^; National Institute of Mental Health (1R01 MH067080-01A2 to C.G.W., 1R01 MH52807); National Institute for Health Research (RP-PG-0610-10100 to D.H.F.); National Institutes Health (MH40799); a VA Merit, REAP, and Medical Center of Excellence Award (principal investigator: R. W. McCarley).
